# Radionuclide therapy of hepatocellular carcinoma

**DOI:** 10.2349/biij.2.3.e40

**Published:** 2006-07-01

**Authors:** FX Sundram

**Affiliations:** Nuclear Medicine and PET/CT Centre, Subang Jaya Medical Centre, Subang Jaya, Selangor, Malaysia

**Keywords:** Hepatocellular carcinoma, iodine-131, lipiodol, rhenium-188, yttrium-90 microspheres

## Abstract

Hepatocellular carcinoma (HCC) is a malignant tumour of the hepatocyte. It is a common malignancy worldwide and causes almost half a million deaths annually. Asia is a high risk area. Although surgery (hepatectomy or liver transplantation) is the main form of curative treatment, the majority of patients are not eligible for surgery due to extent of tumour and dysfunction of liver. Radiopharmaceuticals used for transarterial treatment of HCC were Yttrium-90 microspheres, Iodine-131 lipiodol, Rhenium-188 lipiodol, and Holmium-166 Chitosan complex. Yittrium-90 microspheres are glass or resin microspheres of mean sphere diameter of 20 to 30 micrometre. The activity administered was about 4 GBq. Reported response rate was about 20%, and median survival was 54 weeks. On inoperable tumours, reported objective response of I-131 lipiodol was 40 to 70%, and median survival was six to nine months. It showed efficacy similar to TACE. In adjuvant treatment following curative resection of HCC, reported three year survival was 86% compared with 46% for the control group. The administered activity in both adjuvant and inoperable HCC was about 2 GBq (55 mCi). Rhenium-188 lipiodol is a new radioconjugate, and using it we treated 70 patients with inoperable HCC. This treatment was a part of a multi-centre trial sponsored by the International Atomic Energy Agency. Partial response was obtained in 17% of cases, while 49% had stable disease at three months, and 34% showed disease progression. In terms of survival, 19% survived one year, 60% for six months, and 90% for three months. The mean activity was about 4.6 GBq (124 mCi). This method was safe and free from adverse effects.

## INTRODUCTION

Hepatocellular carcinoma (HCC) is a common malignancy worldwide. It is a major cause of death from cancer in East Asia, especially China, Japan, Korea, Taiwan, and Singapore. Sub-Saharan Africa, particularly Zimbabwe, Ethiopia, and Mozambique faces a similar problem [1].

Surgical resection is generally accepted as the first choice treatment for HCC. Only about 20% of all patients, however, have resectable HCC at initial presentation, due to its multi-focal nature and frequent association with cirrhosis [2]. Treatment of inoperable HCC is mainly palliative. Many non-surgical treatment modalities have been developed and used for the treatment of HCC. These include percutaneous ethanol injection (PEI), cryotherapy, radiofrequency ablation (RFA), systemic chemotherapy, transarterial chemoembolisation (TACE), hormonal therapy, immunotherapy, external radiotherapy, and radionuclide therapy.

PEI and RFA techniques have shown some success in the treatment of small HCCs [4-6]. The use of cryotherapy, systemic chemotherapy, hormonal therapy, immunotherapy, and external beam radiotherapy for the treatment of HCC are either ineffective or have met with limited success [7-9]. Hepatic arterial blood flow can be obstructed by the injection of gelfoam or by placing metallic coils. Flow obstruction can be combined with injection of a mixture of lipiodol and chemotherapy drugs, also known as TACE. TACE is widely used for the treatment of HCC [10], with the caveat that results are better in well-selected patients [11]. A randomised study by Raoul and co-workers has shown a markedly better tolerance for I-131 lipiodol than for TACE with equal long term outcome [12]. Radionuclide therapy has been utilised for palliative treatment of inoperable HCC and for adjuvant therapy following curative resection of HCC.

## PALLIATIVE RADIONUCLIDE THERAPY FOR INOPERABLE HCC

The radionuclide is usually delivered via the hepatic intra-arterial approach. Iodine-131 (I-131) lipiodol, Yttrium-90 (Y-90) microspheres, and Rhenium-188 (Re-188) lipiodol are some of the radiopharmaceuticals that have been utilised. Monoclonal antibodies, such as, anticarcinoembryonic antigen and antiferritin labelled with Y-90 and I-131 have also been utilised and are usually given parenterally. Other approaches, such as, ultrasound-guided percutaneous intra-tumoral injection of Y-90 microspheres have been used. Tian *et al* [13] treated 27 patients with HCC and six patients with liver metastases using this approach, and they found encouraging results. In their study, most of the patients failed other treatment modalities. Twenty-seven patients were still alive 12 to 32 months after treatment, with 90.6% of the tumour foci becoming smaller. The serum α-fetoprotein (AFP) levels were normalised in 10 out of 13 patients. Doses in the range of 282 Gy to 757 Gy were delivered to the tumours.

## I-131 LIPIODOL

I-131 emits both β and γ rays. The average energy of the β rays emitted by I-131 is 181 keV and that of γ rays is 364 keV. It is, therefore, suitable for both therapeutic and imaging purposes. The physical half-life of I-131 is 8.05 days. Lipiodol is an iodised ethyl ester of the fatty acids of poppy seed oil containing 38% of iodine by weight. It is known to selectively localise in HCC following intra-arterial hepatic administration.

Many studies have reported encouraging results with the use of I-131 lipiodol for the treatment of unresectable HCC [14-17]. The activity of I-131 lipiodol administered ranges from 740 MBq to 6,220 MBq, either in single or multiple treatments. Where the hepatic tumours were large, fractionated doses of I-131 lipiodol were given at four-day intervals [14]. Repeat treatment can be given at intervals of 8 to 12 weeks. The objective response rates, in terms of radiological regression of tumour and/or reduction in serum α-fetoprotein levels, range from 40% to 70% [12,14]. Overall, the response of HCC to intra-arterial I-131 lipiodol appears to be dependent on the size of the tumour and the magnitude of the treatment dose delivered transarterially. The response was poorer with increasing size of tumour and lower administered treatment dose. This is to be expected from basic radiation dosimetry principles, which relate cell death to radiation dose absorbed by a tumour.

The efficacy of hepatic intra-arterial I-131 lipiodol for the treatment of HCC is comparable to TACE. I-131 lipiodol appears to be better tolerated than TACE, with fewer side effects experienced by the patients. Radionuclide therapy is, therefore, a reasonable alternative to TACE for the treatment of unresectable HCCs. Two studies have compared the efficacy of hepatic intra-arterial I-131 lipiodol therapy with TACE. In the study by Bhattacharya *et al*, 69 patients with unresectable HCC received hepatic intra-arterial epirubicin-lipiodol emulsion and 26 patients received hepatic intra-arterial I-131 lipiodol [16]. The survival benefit at 6 and 12 months for either modality was comparable. The actuarial survival at 6, 12, and 24 months was 40%, 25%, and 6%, respectively, with epirubicin-lipiodol, and 58%, 25%, and 0%, respectively, with I-131 lipiodol. Both groups showed acceptable toxicity, such as, mild nausea, fever, and abdominal pain. In a French prospective randomised trial [12], 142 patients were randomised to receive either intra-arterial injection of I-131 lipiodol (73 patients) or chemoembolisation (69 patients). It was found that in terms of patient survival and tumour response, both modalities showed similar efficacy in the treatment of HCC. The overall survival rates at six months, one, two, three, and four years were 69%, 38%, 22%, 14%, and 10%, respectively, in the I-131 lipiodol group and 66%, 42%, 22%, 3%, and 0%, respectively, in the chemo-embolisation group. Tolerance of I-131 lipiodol was significantly better with three severe side effects noted in the I-131 lipiodol group and 29 in the chemoembolisation group (p <0.001).

There is also a role for I-131 lipiodol therapy in some patients with portal vein thrombosis where TACE is generally contraindicated. I-131 lipiodol does not modify arterial flow and appears feasible in some patients with portal vein thrombosis. In a French randomised study [15], 14 HCC patients were randomised to I-131 lipiodol therapy and 11 HCC patients to medical support consisting of tamoxifen (five patients), intravenous 5 fluorouracil (one patient), and non-steroidal anti-inflammatory drugs or corticosteroids (five patients). The survival rates at three, six, and nine months were 71%, 48%, and 7%, respectively, for the treatment group and 10%, 0%, and 0%, respectively, for the control group. Overall, the tolerance was excellent in the treated group.

In general, intra-arterial I-131 lipiodol treatment of HCC is well-tolerated [12]. Some reported side effects for this treatment include fever, mild abdominal pain, nausea, elevation of transaminases, and radiation hepatitis [14]. Most of the side-effects are, however, mild and tend to resolve with minimal or no intervention. Pre-treatment with Lugol’s iodine will result in adequate thyroid uptake blockage. There may be a possible role for the use of both radionuclide therapy and TACE together for palliative treatment of HCCs as there may be a synergistic effect. Data on this form of combination therapy are, however, lacking and further studies are awaited.

## Y-90 MICROSPHERES

Y-90 is a stronger pure β emitter than I-131. It emits β rays with an average energy of 935 keV and has a half-life of 64 hours and a maximum penetration or β range of 11 mm. Y-90 does not emit γ rays and is not optimal for imaging purposes. Y-90 can be embedded in insoluble, non-biodegradable glass or resin microspheres (mean diameters of 25 µm to 35 µm). Administration of the Y-90 microspheres via the intra-hepatic, intra-arterial route will result in the deposition of the glass or resin microspheres in the tumour terminal vasculature. HCCs have a relatively greater arteriolar density compared with the normal liver and are predominantly supplied by the hepatic artery rather than the portal venous system. This will result in a three-fold or greater radiation dose in the tumour nodules relative to the normal liver [18]. Y-90 microspheres can deliver a higher radiation dose to the liver tumour compared with I-131 lipiodol. Y-90 microspheres can be safely administered via intra-arterial injection to patients with HCC and underlying cirrhosis at a dose of 100 Gy to the liver [19]. With this technique, an initial angiography scout dose with Tc-99m macroaggregated albumin or other similar agent is required to document the presence of significant porto-systemic shunting in the presence of portal hypertension. Y-90 microspheres are generally not given when the lung uptake is >15%, which indicates significant porto-systemic shunting. Extrahepatic shunting is the main limitation to this form of therapy.

This form of therapy appears to be safe. Andrews *et al* conducted a phase I dose escalation study using intra-arterial Y-90 microspheres with estimated whole liver nominal absorbed doses ranging from 50 Gy to 150 Gy [20]. They did not find any haematologic, hepatic, and pulmonary toxicity during a mean follow-up period of up to 53 months. Reversible gastritis and duodenitis were encountered in four patients. Other reported side effects associated with this form of treatment included fever, elevation of liver enzymes and bilirubin, and gastrointestinal toxicities, such as, ulcers, ileus, and nausea. Most of the side effects did not require treatment and resolved spontaneously. There was one reported case of death resulting from radiation pneumonitis. This patient, however, had 39% pulmonary shunting, and it is questionable whether the patient should have been eligible for treatment.

Several studies have reported the use of Y- 90 microspheres for the treatment of HCC [21-22]. The activity of Y-90 microspheres administered ranged from 1554 MBq to 5,000 MBq for initial treatment with cumulative activity of up to 13,000 MBq from repeated treatments [23,24]. Using the intra-arterial approach of administering the Y-90 microspheres, it is possible to deliver radiation doses of up to 748 Gy to the tumour at a single treatment session and cumulative tumour radiation doses of up to 1,580 Gy [21]. An objective response rate of 80%, in terms of reduction of serum α-fetoprotein levels, has been reported [21]. Other authors have reported an objective response rate of 20% in terms of radiological regression of tumour [22]. Median survival of 9.4 months to 54 weeks has been achieved with the use of Y-90 microspheres [21-25]. Interestingly, Lau *et al* reported four patients whose tumours were converted into resectable ones and these underwent resection [21]. The disadvantage of this treatment is the high cost and the need for two hepatic angiograms.

## RE-188 LIPIODOL

**Figure 1:**
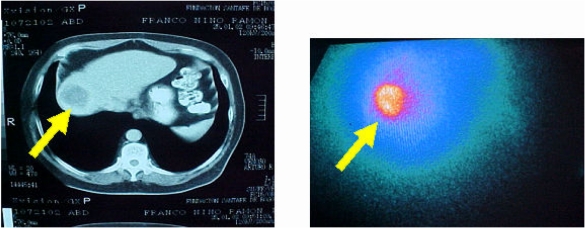
CT scan and Rhenium-188 Lipiodol treatment scan in a patient with HCC.

**Figure 2:**
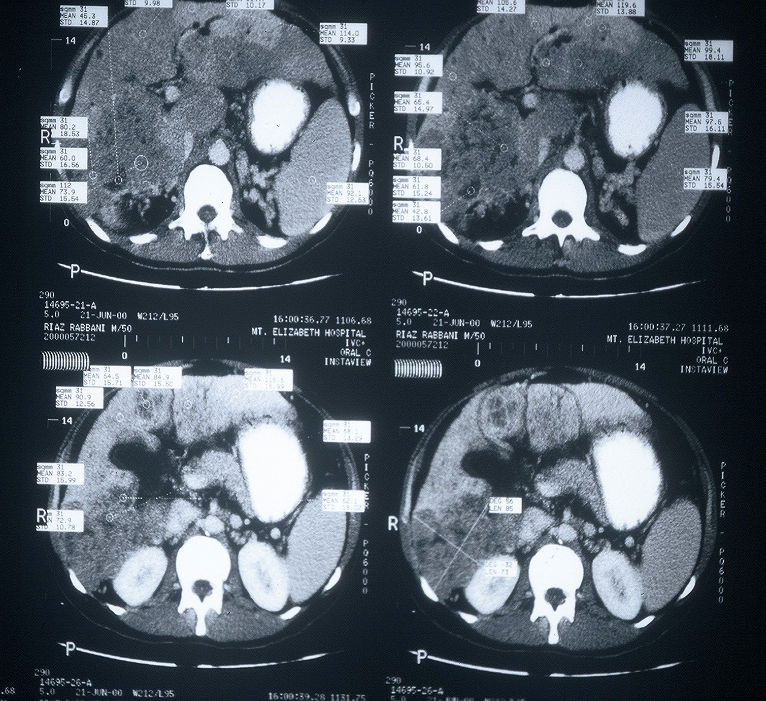
CT scan slices of multifocal liver tumour.

**Figure 3:**
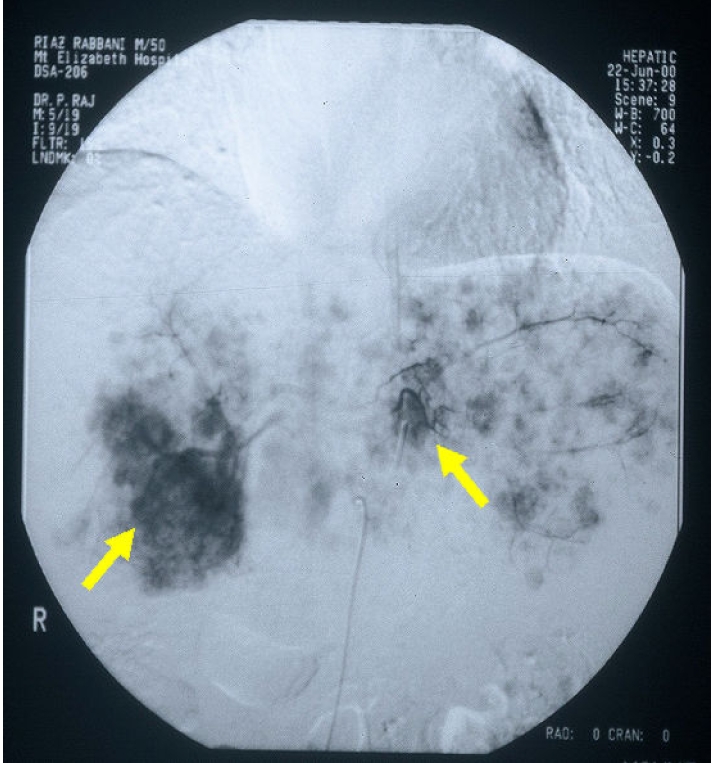
Angiogram image of multifocal tumour.

**Figure 4:**
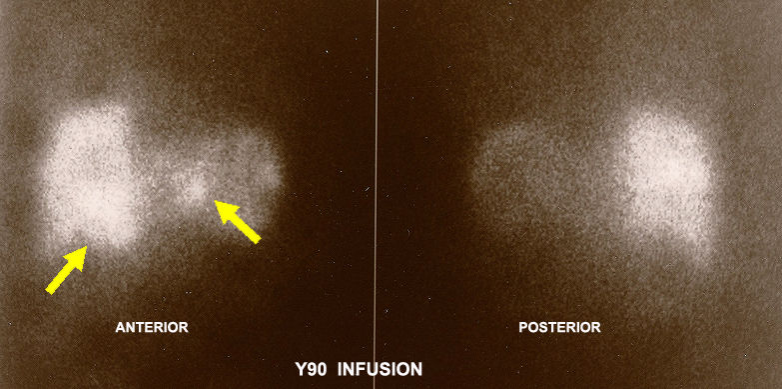
Bremsstrahlung radiation image of Yttrium-90 treatment dose.

Re-188 has recently been used to treat HCCs. It has a physical half-life of 16.9 hours and emits β rays with an average energy of 795 KeV and γ rays of 155 KeV in 15% abundance. Gamma camera imaging for biodistribution studies is possible with this radionuclide. Re-188 was eluted from a W-188/Re-188 generator that has a long and a useful shelf life of several months and, therefore, provides a constant yield of carrier-free Re-188 on a routine basis. This could potentially be cost-saving when compared to the use of other radionuclides and would be particularly useful in the context of treating HCC in developing countries, where incidence is among the highest in the world. The concentrated elute from the tungsten-rhenium generator was heated with 4-hexadecyl 1-2, 9, 9-tetramethyl-4, 7-diaza-1, 10-decanethiol (HDD) in a water bath for one hour to produce a rhenium-HDD complex. The HDD lyophilised kits were obtained from Seoul National University Hospital in Korea. Lipiodol was added and centrifuged to extract the Re-HDD into the lipiodol. This method of preparation was previously described by Jeong *et al* [26] and Lee *et al* [27]. An International Atomic Energy Agency-sponsored multi-centre pilot study using intra-arterial Re-188 lipiodol for the treatment of inoperable HCC showed safety and efficacy of this radioconjugate [28]. Sixteen patients were treated with Re-188 lipiodol in this study. A “scout dose” was given, from which the maximal tolerated dose (MTD) was determined using a specially designed spreadsheet. The MTD is defined as the amount of radioactivity calculated to deliver no more than 12 Gy to the lungs, 30 Gy to the liver, or 1.5 Gy to the bone marrow. These doses have been found to be safe. This method of treatment appears to be safe and well-tolerated at doses up to 7,400 MBq Re-188 lipiodol. The side effects were minimal and included slight elevation of liver enzymes (alanine transaminase and aspartate transaminase) at 24 hours, mild nausea, mild hypochondrial pain, fever, and vomitting. The efficacy of this new radionuclide for the treatment of HCC was confirmed in a larger, multi-centre phase 2 study [29]. Similar results were also reported by Lambert *et al* [30]. Table 1 below gives a summary of the various radionuclides used in the treatment of HCC.Figure 1 shows a CT scan and Re-188 treatment scan. Figure 2 shows CT scans of liver tumour; Figure 3 shows the angiogram image; and Figure 4 shows the Yttrium-90 treatment dose image using the bremsstrahlung radiation.

**Table 1:** A summary of the various radionuclides used in the treatment of HCC

**Radionuclides**	**Half-life (T_1/2_) (hrs)**	**Mean β energy (MeV)**	**γ-energy (keV)**
Yttrium-90 (microspheres)	64	0.94	-
Iodine-131 (lipiodol)	144	0.19	364
Rhenium-188	17	0.79	155
Holmium-166 (chitosan complex)	27	0.67	138
Technetium-99m (macroaggregated albumin)	6	-	140

## RADIO-LABELLED MONOCLONAL ANTIBODIES TO TREAT HCC

There are a few monoclonal antibodies against antigens, such as, CEA, ferritin, and α-fetoprotein, which have some degree of specificity for HCC. Both anti-CEA and antiferritin antibodies have been labelled with I-131 and Y-90 and are administered parenterally for the treatment of HCC. The results have been encouraging, although experience with this method of treatment is limited to only a few centres. The efficacy of this form of treatment may be limited by the fact that the tumour cells are generally heterogeneous and not all the cells express the same antigen. There is also a possibility of developing human-antimouse antibodies with the use of antibodies of murine origin. This will prevent subsequent treatment.

## ADJUVANT TREATMENT WITH I-131 LIPIODOL AFTER CURATIVE RESECTION OF HCC

Resection of HCC is potentially curative, but the recurrence rate is 100% at 5 years. It is hypothesised that the high recurrence rate is due to microscopic metastatic disease or metachronous multicentric tumour present in the remnant liver not detected before and at the time of surgery by the usual imaging methods. Various systemic and locoregional chemotherapy agents have been tried to reduce the rate of recurrence. The overall results show marginal or no significant improvement in disease-free survival. The efficacy of these forms of therapy is also limited by the toxicity of the chemotherapeutic agents. The presence of underlying cirrhosis further limits the ability of the liver remnant to tolerate these agents. In adjuvant therapy, there appears to be a role for radionuclide therapy in improving disease-free and overall survival following curative resection of HCC. Lau *et al* randomised 21 patients to receive one 1,850 MBq dose of intra-arterial I-131 lipiodol and 22 patients to no adjuvant treatment following curative resection of HCC [31]. The median disease-free survival in the treatment and control groups was 57.2 months and 13.6 months, respectively. The three-year overall survival for the treatment and control groups was 86.4% and 46.3%, respectively. In a more recent study, Partensky *et al* reported a median time to detected recurrence of 28 months (range 12 to 62 months) for 28 patients [32]. Each patient was treated with one 1,110 MBq dose of intra-arterial I-131 lipiodol following curative resection of HCC. The overall survival rates were 86% at three years and 65% at five years. In our preliminary study, the six-month disease-free survival rate was 100% and the 12-month disease-free and overall survival rates were 72% and 85%, respectively, for 15 patients who had received I-131 lipiodol adjuvant therapy following curative resection of HCC [33]. This form of adjuvant therapy appears to be safe with no clinically adverse side effects reported. Adjuvant radionuclide therapy may also have a possible role in reducing the rate of tumour recurrence after minimally invasive percutaneous treatment of small HCC, such as, PEI and RFA.

## OTHER METHODS

Some work has been done using P-32 glass micospheres intrarterially, and the results are similar to TACE alone [34]. Re-186 and Re-188 glass microspheres have been effective in animal models, but clinical data are lacking [35]. Ho-166 microspheres and chitosan complex are used in Korea for the treatment of inoperable HCC [36].

## CONCLUSION

Treatment of HCC involves multi-disciplinary collaboration. In cases of multi-segmental or large HCCs, palliative treatment using radionuclide therapy is a viable alternative to TACE. The survival rates are similar for both modalities, but radionuclide therapy appears to be better tolerated with less severe side effects. In rare instances, unresectable tumours may be converted to respectable tumours after radionuclide therapy. Radionuclide treatment in a curative intent may be possible for small HCCs in instances where surgery or percutaneous treatment is not possible due to tumour location or severe concurrent medical illnesses. Adjuvant radionuclide therapy appears to have a promising role in reducing the rate of tumour recurrence after surgery for resectable HCCs and after percutaneous treatment of small HCCs.
